# RICD: A rice *indica *cDNA database resource for rice functional genomics

**DOI:** 10.1186/1471-2229-8-118

**Published:** 2008-11-26

**Authors:** Tingting Lu, Xuehui Huang, Chuanrang Zhu, Tao Huang, Qiang Zhao, Kabing Xie, Lizhong Xiong, Qifa Zhang, Bin Han

**Affiliations:** 1 National Center for Gene Research & Institute of Plant Physiology and Ecology, Shanghai Institutes of Biological Sciences, Chinese Academy of Sciences, PR China; 2 National Key Laboratory of Crop Genetic Improvement and National Center of Plant Gene Research (Wuhan), Huazhong Agricultural University, 430070 Wuhan, PR China

## Abstract

**Background:**

The *Oryza sativa *L. *indica *subspecies is the most widely cultivated rice. During the last few years, we have collected over 20,000 putative full-length cDNAs and over 40,000 ESTs isolated from various cDNA libraries of two *indica *varieties Guangluai 4 and Minghui 63. A database of the rice *indica *cDNAs was therefore built to provide a comprehensive web data source for searching and retrieving the *indica *cDNA clones.

**Results:**

Rice *Indica *cDNA Database (RICD) is an online MySQL-PHP driven database with a user-friendly web interface. It allows investigators to query the cDNA clones by keyword, genome position, nucleotide or protein sequence, and putative function. It also provides a series of information, including sequences, protein domain annotations, similarity search results, SNPs and InDels information, and hyperlinks to gene annotation in both The Rice Annotation Project Database (RAP-DB) and The TIGR Rice Genome Annotation Resource, expression atlas in RiceGE and variation report in Gramene of each cDNA.

**Conclusion:**

The online rice *indica *cDNA database provides cDNA resource with comprehensive information to researchers for functional analysis of *indica *subspecies and for comparative genomics. The RICD database is available through our website .

## Background

Rice is one of the most important crops feeding about half of the world's population. *Indica *and *japonica *are two major Asian cultivated rice subspecies, which show distinct divergence from sequence variations to phenotypic changes [1-3]. The rice *indica *varieties are the most widely cultivated in China, India and most Southeast Asia countries, occupying the largest area of rice production in the world. However, most cDNA resources of the publicly available databases were generated from *japonica *subspecies [4]. Comparison of genomic DNA sequences has showed a large number of variations in genic regions between *indica *and *japonica *[2]. So, collection and analysis of *indica *cDNAs are essential and urgent. Therefore, we collected over 20,000 full-length cDNAs and over 40,000 5' ESTs isolated from various cDNA libraries of two *indica *varieties Guangluai 4 and Minghui 63 during the last few years (Table 1), as part of the National Rice Functional Genomics Project of China [5-7]. The cultivar Guangluai 4 is a typical *indica *variety with the largest growing area during 1968 to 1987 in China [8,9], while the cultivar Minghui 63 is the restore line for lots of planted rice hybrids. Systematic analysis and utilization of the *indica *cDNA clones would be valuable to rice genetics and breeding. Therefore, a dedicated platform is required for search, characterization and retrieval of the *indica *cDNA clones.

**Table 1:** Sources of *indica *cDNA sequences in the RICD database.

	Number of cDNAs	Number of cDNAs matching IRGSP 4*	Number of cDNAs matching TIGR Gene**
Guangluai 4 FL-cDNA	10,081	9,109 (90.4%)	7,680 (76.2%)
Minghui 63 FL-cDNA	12,727	12,317 (96.8%)	8,209 (64.5%)
Guangluai 4 5'EST	21,690	16,504 (76.1%)	8,700 (40.1%)
Minghui 63 5'EST	27,130	21,059 (77.6%)	15,115 (55.7%)

* IRGSP pseudomolecules Build 4.

** TIGR rice gene annotation version 5

RICD was developed to provide a handy way to access all the available *indica *cDNA sources and be integrated with comprehensive information, which includes encoded amino acid sequence information, the mapping information, the protein domain information, the results of similarity search, gene function annotation and so on. Several search and retrieval forms were developed to create a comprehensive data warehouse for rice functional genomics and comparative genomics research.

## Construction and content

### Database architecture

RICD is a relational database, and was developed using MySQL 4.1. The database was implemented on a server running Red Hat Enterprise Linux AS release 4 (×86). The web interface was constructed by using PHP scripts (PHP Version 4.3), and powered by an Apache server (Apache Version 2.0).

### cDNA acquisition

The *Oryza sativa *L. *indica *Guangluai-4 cDNAs were generated from five types of cDNA libraries [7]. The libraries included: (1) Seedlings grown in water for two weeks with a cycle of 16 hours light and 8 hours dark at 30°C; (2) Panicles harvested from rice grown in paddy fields; (3) Root grown in water for two weeks with a cycle of 16 hours light and 8 hours dark at 30°C; (4) Two-day germinated shoots and roots collected when roots reached 1–2 cm long; (5) Two-week seedlings treated individually with various stresses, that is, high-salinity (100 mM NaCl, treated for 20 min, 3, 12, 24, 48 h, 3 days and recovered for 72 h), dehydration (15% PEG-4000, treated for the same time duration as high-salinity), cold (60°C for 1, 12, 24, 48 h, 3 days and recovered for 72 h), heat (45°C, for the same time duration as cold), or immersion under water (for 1, 12, 24, 48 h, 3, 5 days). The *Oryza sativa *L. *indica *Minghui-63 cDNAs were from a normalized whole-life-cycle cDNA library, which was constructed by use of 15 tissues collected from 9 developmental stages [5].

### Sequence analysis

The cDNAs and TIGR rice genes were remapped to IRGSP pseudomolecules Build 4 [3,10] using the GMAP program [11]. Over 90% putative full-length cDNAs of Guangluai 4 and Minghui 63 could be matched to rice IRGSP pseudomolecules, and 76.2% of Guangluai 4 cDNAs and 64.5% of Minghui 63 cDNAs could be matched to TIGR gene loci, respectively. In addition, > 76% 5' ESTs of Guangluai 4 and Minghui 63 were matched to IRGSP pseudomolecules, and > 40% of them could be matched to TIGR gene loci. The cDNAs were annotated according to RAP-DB [12] and TIGR systems [13]. Other sequence analysis was performed as described previously [7].

## Utility

**Figure 1:**
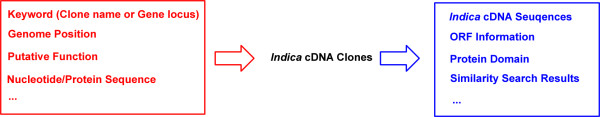
**Query and display of cDNA clones in Rice *Indica *cDNA Database**. The query parts are in red, while the display parts are in blue.

RICD provides an interactive and user-friendly web interface for searching the cDNA clones and retrieving their sequences along with other detailed information (Figure 1). The panel on the right of the main page lists hyperlinks to the different search pages, online tools and web pages describing various information of the database. The cDNA component of the database can be searched by multiple ways as described below.

### BLAST search

The BLAST program (version 2.2) is integrated into RICD for sequence search. Either nucleotide sequence (BLASTN) or protein sequence (TBLASTN) can be used as query to search all *indica *cDNAs or any subset of the database, with an initial cut-off E value as 0.01 [14]. A typical BLAST result page displays cDNAs matching the query, with their clone ID and the sequence alignments.

### Keyword search

The cDNA of the database can be searched directly using clone name, NCBI accession number, RAP gene locus ID, or TIGR gene locus ID. The NCBI accession number commonly starts with the two characters "CT". And two separate 'gene locus ID search' sections, for RAP gene locus ID and TIGR gene locus ID, were given. The querying result will show a list of entries that are linked to the individual cDNA clones.

### Chromosome position search

Two tools were designed for users to look up cDNAs in the rice genome. One shows in table form, naming "Clone list". You can directly enter the appointed region to get a list of cDNAs in the region, each of which can be further entered individually. A separate web page is provided for listing the putative *indica*-specific cDNAs, which cannot be mapped into IRGSP pseudomolecules Build 4. You can also choose a browser tool "mapping view" to visualize the graphic display. The main browser display contains two main parts. On the top are the scales for users to specify a particular region in the genome. On the bottom shows a series of cDNAs in the database, and also RAP-DB and TIGR gene loci. Upon clicking one, users can enter into result display page of the cDNA clone.

### Function search

**Figure 2:**
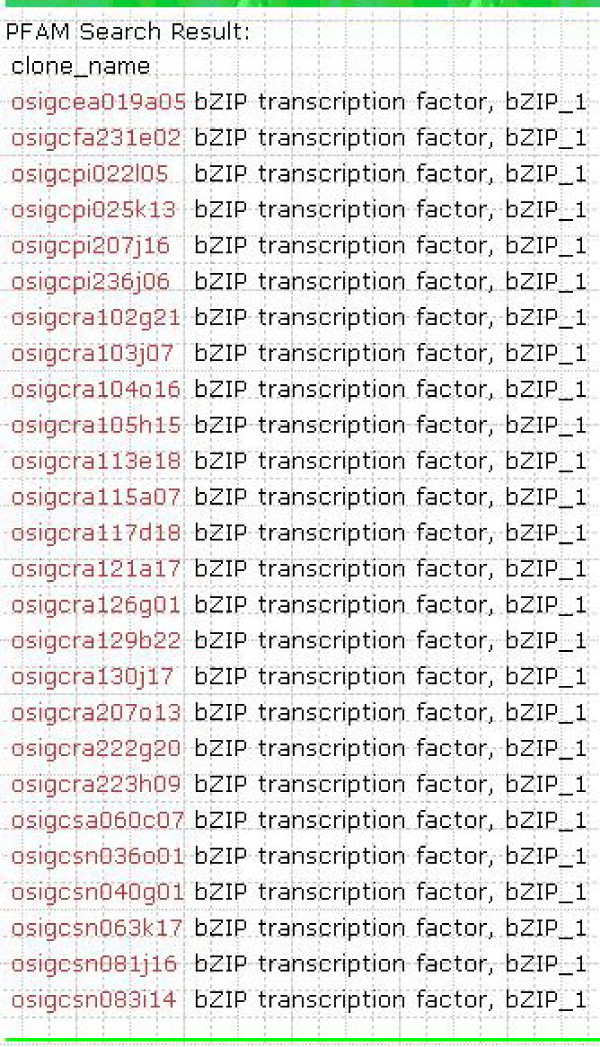
**A example result page of a putative function query**. The figure shows the page displaying Pfam search results for query term "bZIP transcription factor".

To query the cDNA clones, users can also enter a putative function (bZIP transcription factor, receptor protein kinase, and so on) to retrieve the responding cDNA clones (Figure 2). The putative functions rely on the protein domain Pfam descriptions [15] or RAP-DB gene annotations of any individual cDNA annotated.

### cDNA report display

**Figure 3:**
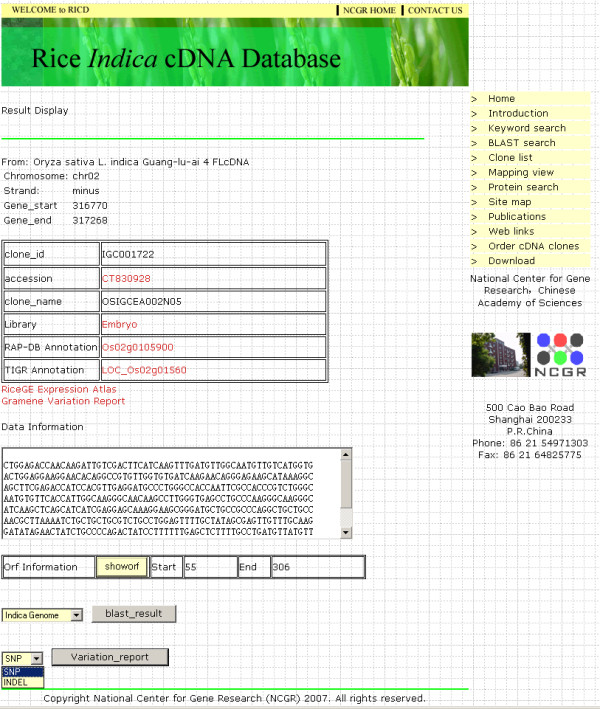
**A typical result display page of a cDNA**. The information of an *indica *cDNA clone includes the clone ID, GenBank Accession, library, genome position, gene annotation, expression atlas, variation report, cDNA sequence, ORF information. SNPs and InDels information and the results of similarity search will be shown after clicking corresponding buttons.

For each cDNA clone, the result display page consists of three sections (Figure 3). The first section provides general information about the cDNA: genome position, clone name, accession number, library, RAP gene locus, TIGR gene locus, expression atlas in RiceGE [16] and variation report in Gramene [17]. The second section provides sequence of the cDNA and its ORF information. The last section includes the information of SNPs and InDels between *japonica *KOME and *indica *NCGR FL-cDNA (full-length cDNA) pairs and the results of similarity search with *japonica *and *indica *genome, KOME FL-cDNA and NCBI non-redundant databases.

## Conclusion

The initial aim of RICD is to provide a convenient way to search and retrieve *incdia *cDNA clone for research community. Via integrating with comprehensive information, it tries to grow to be a platform for broad applications to rice genetics, breeding and comparative genomics. Future expansion plans of the database include recruiting more *indica *cDNA clones, cataloging splice variants and adding gene expression data from *indica *cDNA microarray. The RICD database will be updated frequently if more information becomes available.

## Availability and requirements

The RICD resource can be freely accessed via .

The cDNA clones in the database can be requested for research purpose by contacting Tingting Lu ttlu@ncgr.ac.cn

## Authors' contributions

* The authors wish it to be known that, in their opinion, the first three authors should be regarded as joint first authors. TL designed the structure and layouts of database, provided the majority of annotations of sequences and partly drafted the manuscript. XH participated the designing of database, provided some annotation results and mostly drafted the manuscript. CZ developed the database and its web interface. TH and QZ provided the technical supports and discussion. KX, LX and QZ provided the batch sequences of Minghui-63 variety. BH conceived of the study, and participated in its design and helped to draft the manuscript. All coauthors read and approved the final manuscript.
